# Physical Model of Inclusions Removal at Static Steel–Slag Interface

**DOI:** 10.3390/ma17102244

**Published:** 2024-05-10

**Authors:** Xin Tao, Jianqi Cao, Jia Wang, Xiaonai He, Lingyu Meng, Yongbo Guo, Tao Wang, Dongliang Li, Jinping Fan, Chao Chen

**Affiliations:** 1 College of Materials Science and Engineering, Taiyuan University of Technology, Taiyuan 030024, China; taoxin0290@link.tyut.edu.cn (X.T.); 233501007@csu.edu.cn (J.C.);; 2 School of Metallurgy and Environment, Central South University, Changsha 410083, China; 3 Department of Chemistry & Chemical Engineering, Lyuliang University, Lishi 033001, China

**Keywords:** inclusions, slag–steel interface, motion behavior, water model

## Abstract

Inclusions are one of the important factors affecting the cleanliness of molten steel. The current optimization of inclusion removal methods mainly focuses on promoting inclusions to float to the slag–steel interface so that the inclusions can be absorbed and removed by the refining slag. However, the research on the floating removal of inclusions cannot be carried out directly in the ladle, so methods such as mathematical models and physical models were developed. This article uses silicone oil to simulate the slag layer; polypropylene particles; and aluminum oxide particles to simulate inclusions to establish a water model experiment. By changing the viscosity of silicone oil and the diameter of particles, the factors affecting the movement of inclusions at the slag–steel interface were explored. Based on the water model, a mathematical model of the floating behavior of inclusions at the slag–steel interface was constructed, and parameters such as particle diameter and interfacial tension in the water model experiment were studied by the mathematical model for calculation. Both the mathematical model and the water model experimental results show that after the viscosity of silicone oil increases from 0.048 Pa·s to 0.096 Pa·s, the dimensionless displacement and terminal velocity of the particles decreases. When the diameter of the same particle increases, the dimensionless displacement and terminal velocity increases. The dimensionless displacement of polypropylene particles of the same diameter is larger than that of aluminum oxide particles, and the terminal velocity is smaller than that of aluminum oxide particles. This is attributed to the better overall three-phase wettability of polypropylene particle. When the liquid level increases, the dimensionless displacement and terminal velocity of particles under the same conditions show only slight differences (less than 10%).

## 1. Introduction

Inclusion removal is a crucial step in the steelmaking process, and the removal effect directly affects steel quality [1,2,3,4]. At present, the main methods of inclusion removal include gas stirring in ladles [5,6,7,8,9,10], Ruhrstahl–Heraeus treatment [11,12,13,14], slag washing [15,16,17], bubble flotation [18], removal in tundishes [19,20,21,22], and continuous casting mold [23,24,25,26], among others. These methods promote inclusions to float to the slag–steel interface so that the inclusions are more easily removed by refining slag absorption. The process of inclusion absorption by the top slag can be divided into four steps [27,28,29]: (1) inclusions are transported to the turbulent boundary layer of the steel–slag interface; (2) inclusions are transported through the boundary layer to the steel–slag interface; (3) inclusions separate to the slag; and (4) inclusions are dissolved in the slag phase. Many scholars have studied these four steps separately: for example, step 1 [30,31,32,33,34], step 2 [29,35,36], step 3 [27,37,38,39,40], and step 4 [41,42,43,44,45]. The results show that the separation process of inclusions at the slag–steel interface is the critical link of inclusion absorption by slag [2].

However, it is almost impossible to study the removal of inclusions in liquid steel directly. The main research methods are mathematical and physical models. The mathematical models mainly include the force equilibrium and computational fluid dynamics models. The force equilibrium model based on Newton’s second Law started from the research of K. Nakajima and K. Okamura [37] and studied the force condition of inclusions during the floating process and during the formation of steel film, when the inclusions pass the slag–steel interface. In the following decades, some scholars [46] revised the model, considering the influence of the turbulent bursting phenomenon on the motion of inclusions. Nakajima and coauthors [27,38,39,40] continued to develop this model; the three statuses of inclusions moving at the slag–steel interface were illustrated, and the motion model of liquid inclusions was established. The separation model of aluminum oxide (abbreviated as Al_2_O_3_) inclusion past the slag–steel interface was established by G.N. Shannon et al. [47,48,49,50]. The motion of solid inclusions with different shapes at the slag–steel interface was calculated by Y.L. Zhou et al. [51]. They also considered the interface deformation resistance caused by the slag–steel interface deformation around the inclusions. The original framework of the mathematical model has been developed and applied to more complex slag systems [52]. The computational fluid dynamics model mainly coupled solid and multiphase flow, and the motion trajectory of inclusions at the slag–steel interface can be observed and analyzed [42]. Recently, CFD models coupled with flow–solid interaction [53,54] or the use of the dynamic overset grid technique [55] were developed to study the inclusion motion and separations.

The physical models are mainly based on the similarity principle, using appropriate material to simulate liquid steel, molten slag, and inclusions and recording the floating of “inclusions” and the movement process at the “slag–steel” interface to study the movement behavior of inclusions in reality [56,57,58,59,60,61]. The water model study on inclusion movement at the interface can be dated back to the work by Ferchichi and Duval [56]. Glycerol and silicone oil were used to simulate heavy and light phases, respectively, and millimetric polymer spheres were used to simulate the inclusion particles. A high-speed camera was used to record the rising process of particles approaching the interface. The focus of their study was on the examination of the hydrodynamic correction factor and effect of the dimensionless numbers on drainage time. In their work, the glycerol (simulate the liquid steel) showed an even higher viscosity than the silicone oil (simulate the slag phase), which does not correspond to the steel–slag properties. In 2016, Liu et al. [58] utilized silicone oil and hollow Al_2_O_3_ particles to simulate the slag layer and inclusions, respectively. Two kinds of oil with different viscosities were studied. However, their main focus was on the validation of mathematical models. In 2017, Zhou et al. [59] studied the effects of inclusion geometry (shape and size) and slag properties (viscosity and interfacial tension) on the separation process. Three different kinds of paraffin wax particles (octahedral, plate-like, and spherical) and three different oils (kerosene, bean oil, and pump oil) were used to model inclusions and slags, respectively. Zhu et al. [60] studied the cluster inclusions motion at the steel–slag interface by water models. The cluster particles were fabricated by three-dimensional printing technology. Recently, Nishibata et al. [61] performed a comprehensive water model to study the particle agglomeration breakup and engulfment at the steel–slag interface. The hydrophobic and hydrophilic polymethylmethacrylate (PMMA) particles were used to study two kinds of inclusions.

However, the influence of the height of the liquid level and the type of particles on the movement of the “inclusions” at the slag–steel interface have not been thoroughly studied in the literature. In this paper, by establishing a water model, the influence of the height of the liquid level, the type and size of the inclusion particles, and the viscosity of the slag (silicone oil) on the movement of the inclusions at the slag–steel interface are explored to predict the factors affecting the movement of inclusions at the slag–steel interface in the process of steelmaking.

## 2. Experimental Method

### 2.1. Water Model Experiment

#### 2.1.1. Similarity Principle

(1) Static similarity

The inclusions float up to the slag–steel interface, and whether they can pass the steel–slag interface depends on the interaction and wettability between the inclusions, slag, and molten steel. Therefore, it is necessary to ensure the static similarity of the three phases when establishing the system.

In this experiment, water is used to simulate molten steel, so the material density of the simulated slag should be smaller than that of water. Therefore, several oils with a lower density than water are selected as alternatives. After comparison, it is found that the interface between silicone oil and water is obvious, so silicone oil is selected as the simulation material of slag. The interfacial tension between non-oily material and oil is small, especially for the polymer [57]. The wetting effect of oil on such particles is better, which can be used to simulate the wetting of slag and absorption of oxide inclusion in the smelting process. Therefore, in this experiment, polypropylene (abbreviated as PP) and hollow Al_2_O_3_ particles are adopted to simulate the inclusion in the steel. The motion process of the particles floating up to the oil layer and the motion behavior at the water–oil interface could simulate the motion process of the non-metallic inclusions floating up to the top slag and the motion behavior at the steel–slag interface.

(2) Dynamic viscosity similarity

To ensure that the water–oil interface has suitable interface properties and that the dynamic viscosity of silicone oil is similar to that of actual slag:

(1) $$\frac{\mu_{w}}{\mu_{\mathrm{st}}}=\frac{\mu_{o}}{\mu_{\mathrm{sl}}}$$

where, μ_w_, μ_o_, μ_st_, and μ_sl_ are the dynamic viscosity of water, silicone oil, molten steel, and slag, respectively.

According to references [62,63,64,65,66], the dynamic viscosity of liquid slag is about 0.02~0.3 Pa·s, and the viscosity of molten steel is about 2~6.5 × 10^−3^ Pa·s. At the temperature of 293 K, the dynamic viscosity of water is about 1 × 10^−3^ Pa·s, so the dynamic viscosity of the simulated silicone oil calculated from Equation (1) ranges between 0.0031 and 0.15 Pa·s.

The viscosity of silicone oil used in this experiment is 0.048 Pa·s and 0.096 Pa·s, which can satisfy the requirements of similar dynamic viscosity. In addition, the oil viscosity in the studies are 0.023 and 0.072 Pa·s by Liu et al. [57]; 0.0027, 0.0592, and 0.2737 Pa·s by Zhou et al. [59]; 0.00648 Pa·s by Zhu et al. [60]; and 0.0018 Pa·s by Nishibata et al. [61], respectively.

#### 2.1.2. Experimental Equipment and Materials

**Figure 1:**
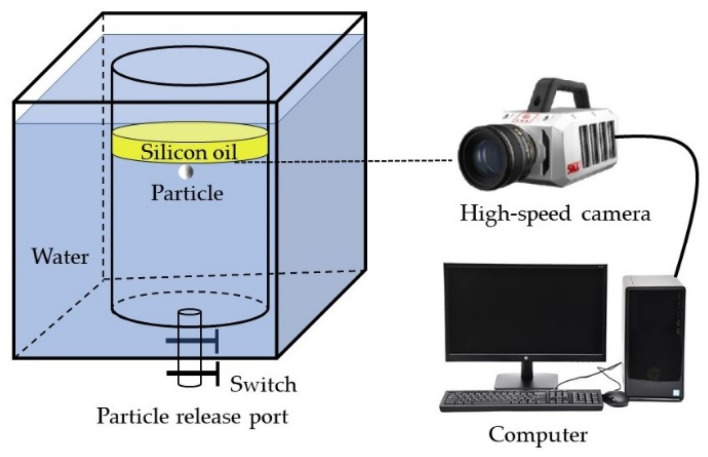
Schematic diagram of experimental equipment.

The container used in this experiment is composed of acrylic. The inside is a cylindrical container with a diameter of 445 mm and height of 600 mm and a hole with a diameter of 10 mm at the center of the bottom, which acts as a particle release port. To eliminate the refraction phenomenon, the rectangular container with a 600 mm edge is surrounded by the cylinder. The image-recording equipment that was used in the experiment is the FuHuang Agile Device Revealer 5KF20S 3000 high-speed camera (which from Hefei Fu huang junda gaoke company in Hefei, China) and its motion analysis software. The shooting frame rate is 100, and the shooting time is 10 s. The schematic diagram of the experimental equipment is shown in Figure 1. The materials used in the experiment and their physical properties are listed in Table 1.

#### 2.1.3. Experimental Method

The liquid level height in the experimental container is 30 or 50 cm, and the thickness of silicone oil is 5 mm. Polypropylene (abbreviated as PP) particles and Al_2_O_3_ particles of different sizes were released from the bottom of the container, and a high-speed camera was used to capture the movement of the particles at the interface. The particle displacement data are processed through motion analysis software to obtain the dimensionless displacement and velocity changes of the particles.

### 2.2. Mathematical Model of Inclusions Moving at Steel–Slag Interface

In previous research, we established a mathematical model for the movement and floating of inclusions at the slag–steel interface and verified the accuracy of the mathematical model [52]. This model can analyze the force during the floating process of inclusions and predict the final residence position of inclusions at the slag–steel interface. Therefore, the water, oil, particles, and interfacial tension parameters in the water model experiment can be substituted into the mathematical model for calculation, and the results of the water model experiment can be compared and analyzed. The specific details of the mathematical model are described in the following sections.

#### 2.2.1. Model Assumptions

The basic assumptions of the mathematical model of the motion behavior of inclusions at the slag–steel interface are as follows:

(1) Inclusions are spherical with constant volume;
(2) No chemical reaction occurs between phase interfaces;
(3) All fluids are incompressible and isothermal;
(4) The slag phase exists in the form of a liquid phase;
(5) The slag–steel interface is smooth;
(6) The movement process of inclusions depends on buoyancy, rebound force, drag force, and additional mass force;
(7) Surface tension is uniform at the interface.

**Figure 2:**
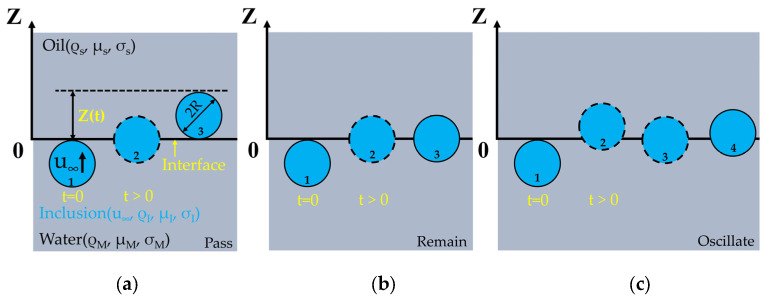
Three motion conditions of inclusions at the slag–steel interface: (**a**) pass; (**b**) remain; (**c**) oscillate.

As shown in Figure 2, the movement of inclusions at the slag–steel interface can be divided into three types: passing, remain, and oscillate [52]. As shown in Figure 2a, the movement path of the particle is 1 → 2 → 3, and the final displacement is 2R. In this case, it is believed that when Z ≥ 2R, the particles pass through the water–oil interface and enter the oil layer. As shown in Figure 2b, the movement path of the particles is 1 → 2 → 3. The particle’s final displacement distance is between 0 and 2R, and there is no rebound or rise again, which is considered to be the remain type. As shown in Figure 2c, the particle motion path is 1 → 2 → 3 → 4. The process in which the particles rebound and then rise obviously is considered to be oscillate type, and the final displacement distance is also between 0 and 2R.

#### 2.2.2. Motion Model of Inclusions

**Figure 3:**
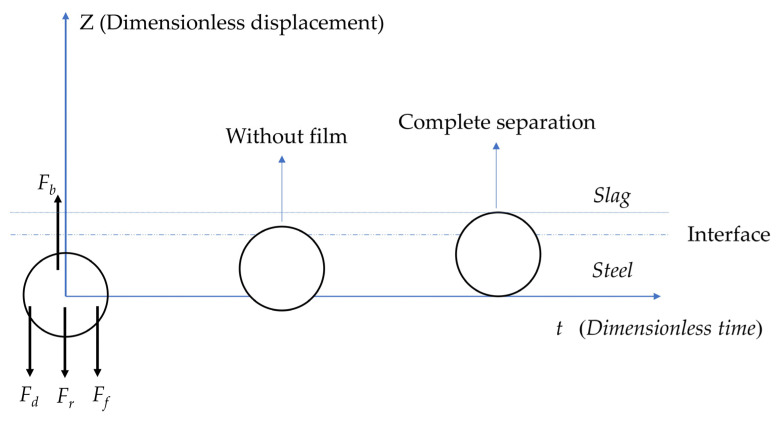
Schematic diagram of the inclusion movement.

The schematic diagram of the movement of inclusions at the slag–steel interface is shown in Figure 3. When inclusions approach the slag–steel interface, four forces act on the inclusions. These four forces are buoyancy force $F_{b}$ rebound force $F_{r}$, drag force $F_{d}$, and additional mass force $F_{f}$. The buoyancy force always appears upward, while the rebound force, additional mass force, and drag force can appear upward or downward depending on the movement behavior of the inclusions at the interface.

According to Newton’s second law:

(2) $$\frac{4}{3}\pi R_{I}^{3}\rho_{I}\frac{d^{2}Z}{dt^{2}}=F_{b}-F_{d}-F_{r}-F_{f}$$

where Z is the displacement of the inclusion; t is the movement time of the inclusion; $R_{I}$ represents inclusion diameter; and $\rho_{I}$ represents inclusion density.

The expression of buoyancy $F_{b}$ is as follows:

(3) $$F_{b}=\frac{4}{3}\pi R_{I}^{3}g(\rho_{s}\cdot A(Z^{*})-\rho_{I})$$

(4) $$A(Z^{*})=\frac{1}{4}(\frac{\rho_{m}}{\rho_{s}}-1)Z^{*3}-\frac{3}{4}(\frac{\rho_{m}}{\rho_{s}}-1)Z^{*2}+\frac{\rho_{m}}{\rho_{s}}$$

where $\rho_{s}$ is the density of the slag; $Z^{*}$ represents dimensionless displacement (actual displacement/particle radius); and $\rho_{m}$ is the density of molten steel. $A(Z^{*})$ represents the density change term, indicating the degree of inclusions entering the slag layer.

The expression of additional mass force $F_{f}$ is as follows:

(5) $$F_{f}=\frac{2}{3}\pi R_{I}^{3}\rho_{s}\cdot A(Z^{*})\cdot g\frac{d^{2}Z^{*}}{dt^{{*}^{2}}}$$

The expression of rebound force $F_{r}$ is as follows:

(6) $$F_{r}=2\pi R_{I}\sigma_{\mathrm{MS}}B(Z^{*})$$

where $\sigma_{\mathrm{MS}}$ is the interfacial tension between metal (steel) and slag; $B(Z^{*})$ is replaced by the following expression:

(7) $$Β(Z^{*})=Z^{*}-1-\cos\theta_{\mathrm{IMS}}$$

where $\cos\theta_{\mathrm{IMS}}$ is the overall wettability of the steel–slag inclusion system, and the expression is as follows:

(8) $$\cos\theta_{\mathrm{IMS}}=\frac{\sigma_{\mathrm{IM}}-\sigma_{\mathrm{IS}}}{\sigma_{\mathrm{MS}}}$$

where $\sigma_{\mathrm{IM}}$ is the interfacial tension between inclusions and molten steel and $\sigma_{\mathrm{IS}}$ is the interfacial tension between inclusions and slag.

The expression of drag force $F_{d}$ is as follows:

(9) $$F_{d}=6\pi AR_{I}\mu_{S}\cdot C(Z^{*})\cdot \sqrt{R_{I}g}\frac{dZ^{*}}{dt^{*}}$$

where $t^{*}$ is the dimensionless movement time of the inclusion, and A is a function of k, which is related to the viscosity of inclusions and molten steel. $\mu_{S}$ is the viscosity of the slag, and $C(Z^{*})$ is expressed by the following formula:

(10) $$A=\frac{2+3k}{2(1+k)}$$

(11) $$C(Z^{*})=\left(\frac{\mu_{M}}{\mu_{S}}-1\right)Z^{*2}-2\left(\frac{\mu_{M}}{\mu_{S}}-1\right)Z^{*}+\frac{\mu_{M}}{\mu_{S}}$$

Substituting Equations (3), (5), (6) and (9) of the buoyancy force, additional mass force, rebound force, and drag force into Equation (2), the following dimensionless displacement equation is written as:

(12) $$\frac{d^{2}Z^{*}}{dt^{*2}}=2\frac{\rho_{s}\cdot A(Z^{*})-\rho_{I}}{\rho_{s}\cdot A(Z^{*})+2\rho_{I}}-3\cdot D(Z^{*})\cdot B(Z^{*})-\frac{9}{E(Z^{*})}\cdot C(Z^{*})\cdot \frac{dZ^{*}}{dt^{*}}$$

where $D(Z^{*})$ and $E(Z^{*})$ can be expressed by the following formula:

(13) $$D(Z^{*})=\frac{\sigma_{\mathrm{MS}}}{\;R_{I}^{2}\left(\rho_{s}\cdot A(Z^{*})+2\rho_{I}\right)g}$$

(14) $$E(Z^{*})=\frac{\sqrt{R_{I}^{3}g}(\rho_{s}\cdot A(Z^{*})+2\rho_{I})}{\;\mu_{S}}$$

Finally, the dimensionless expressions of displacement, time, velocity, and acceleration can be obtained:

(15) $$Z^{*}=\frac{Z}{R_{I}}$$

(16) $$t^{*}=t\sqrt{\frac{g}{R_{I}}}$$

(17) $$\frac{dZ^{*}}{dt^{*}}=\frac{1}{\sqrt{gR_{I}}}\frac{\mathrm{dZ}}{\mathrm{dt}}$$

(18) $$\frac{d^{2}Z^{*}}{dt^{*2}}=\frac{1}{g}\frac{d^{2}Z}{dt^{2}}$$

**Figure 4:**
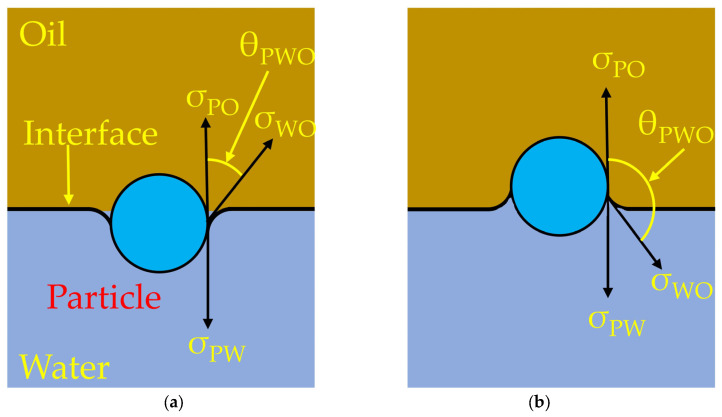
The relationship between σ_pw_, σ_po_, σ_wo_, and θ_pwo_. (**a**) cosθ_PWO_ > 0, (**b**) cosθ_PWO_ < 0.

The overall wettability diagram of the water–oil system is shown in Figure 4, and the expression is given as:

(19) $$\cos\theta_{\mathrm{PWO}}=\frac{\sigma_{\mathrm{PW}}-\sigma_{\mathrm{PO}}}{\sigma_{\mathrm{WO}}}$$

The relationship between σ_pw_, σ_po_, σ_wo_, and θ_pwo_ is shown in Figure 4. There are two wetting states between slag and inclusion (oil and particle), as shown in Figure 4a; cosθ_PWO_ > 0, which means the inclusion shows a good wettability for slag. If cosθ_PWO_ < 0, as shown in Figure 4b, this means that the inclusion shows a poor wettability for slag.

Relevant data on hollow Al_2_O_3_ particles can be found in the literature [67], but data on polypropylene particles are missing, so reasonable estimation is needed. According to polypropylene material’s hydrophobic and oleophilic characteristics [68,69], relevant parameters found in the literature and estimated are listed in Table 2.

In order to solve Equation (12), the Runge–Kutta method is used [70]. By using the ode45 function in MATLAB2018b, the relationship between the displacement, velocity, and acceleration of the inclusions as a function of time are solved; finally, the forces of the inclusions during the floating process and the final residence position of the inclusions at the slag–steel interface are obtained. The water, oil, particles, and interfacial tension parameters in the water model experiment are substituted into the mathematical model for calculation and compared with the water model results.

## 3. Results and Discussion

### 3.1. Mathematical Model Verification

**Figure 5:**
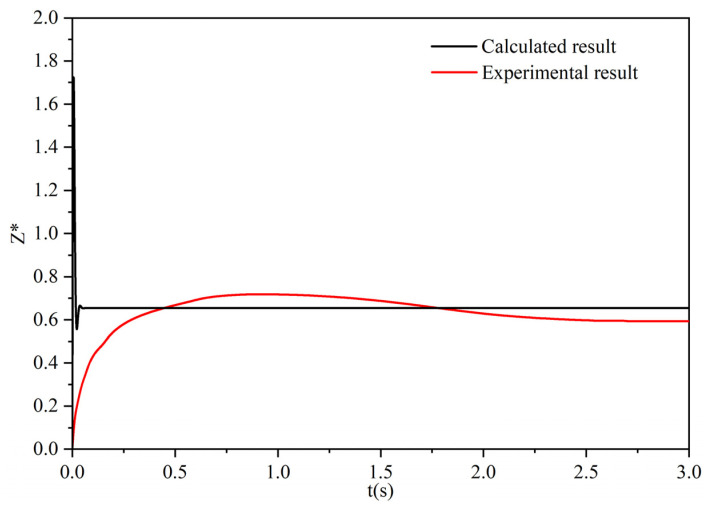
Comparison of experimental result and calculated result of dimensionless displacement.

In order to verify the accuracy and reliability of the mathematical model, the calculation results and experimental results of hollow Al_2_O_3_ particles with a diameter of 2.1 mm are shown in Figure 5. The calculation results show that after the particles contact the water–oil interface, they first float up for a certain distance and then rebound; then, they float up again until they stay at the water–oil interface, which is in good agreement with the experimental results. Moreover, the final dimensionless displacements of the calculated results and experimental results are 0.655 and 0.594, respectively, which is in good agreement [52].

### 3.2. Calculation Results of the Mathematical Model

#### 3.2.1. Analysis of Force during Particle Floating

**Figure 6:**
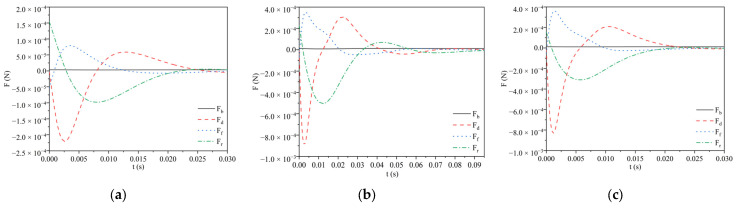
Force analysis diagram of the water–oil interface of two kinds of particles: (**a**) 2 mm polypropylene particle; (**b**) 3 mm polypropylene particle; (**c**) 2 mm Al_2_O_3_ particle.

Figure 6 shows the force analysis diagram of the two particles at the water–oil interface with the viscosity of silicone oil at 0.096 Pa·s. Figure 6a,b show the force analysis diagram of polypropylene particles with diameters of 2 mm and 3 mm, respectively. It can be seen from the figure that it takes about 0.03 s for a polypropylene particle with a diameter of 2 mm to reach the force equilibrium, while it takes about 0.09 s for a polypropylene particle with a diameter of 3 mm to reach the force equilibrium. The time is extended, that is, with the increase in the diameter of the polypropylene particle, the time for the particle to reach the force balance in the process of motion is longer. Figure 6c shows the force analysis diagram of an Al_2_O_3_ particle with a diameter of 2 mm. By comparing Figure 6a,c, it can be seen that the polypropylene particle and Al_2_O_3_ particle with the same diameter take almost the same time to reach the force equilibrium in the process of motion, which is about 0.03 s. Hence, it can be inferred that the time required for particles to reach force equilibrium at the water–oil interface correlates with particle size rather than particle type.

#### 3.2.2. Analysis of Calculation Results of Dimensionless Displacement of Particles

In this calculation model, only the vertical force is considered when the particle floats, so it is believed that the upward floating process of the particle is only the vertical displacement. The dimensionless displacement of a particle is the ratio of the particle’s actual displacement to the particle’s radius. The final states of particles at the steel–slag interface can be divided into three types [52]: pass, remain, and oscillate.

**Figure 7:**
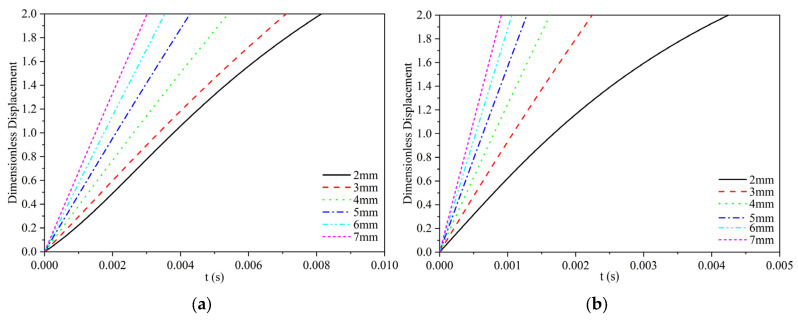
The calculation results of dimensionless displacement curves of two particles with different particle sizes at the water–oil interface when the viscosity of silicone oil is 0.048 Pa·s: (**a**) polypropylene particles; (**b**) Al_2_O_3_ particles.

When the viscosity of silicone oil is 0.048 Pa·s, the dimensionless displacement of polypropylene and Al_2_O_3_ particles with different sizes at the water–oil interface is shown in Figure 7. Figure 7a,b shows the dimensionless displacement of polypropylene and Al_2_O_3_ particles with different particle sizes of 2–7 mm, respectively. It can be seen that both particles can pass the water–oil interface. From the perspective of particle size, the larger the particle diameter, the shorter the time required to cross the water–oil interface. This tendency of shortening of the time is reduced with an increase in diameter. In terms of particle types, the time required for Al_2_O_3_ particles to pass the water–oil interface is shorter than that for polypropylene particles.

**Figure 8:**
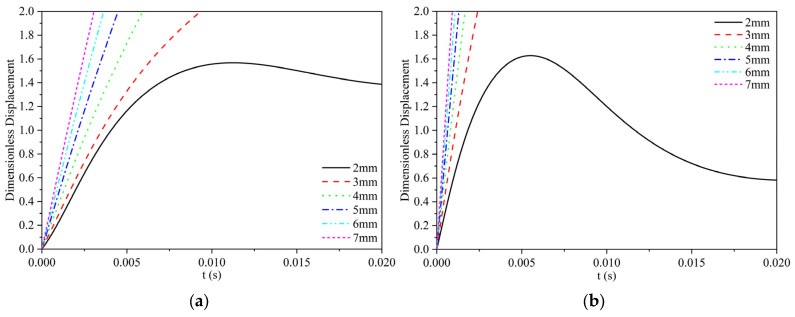
The calculation results of dimensionless displacement curves of two particles with different particle sizes at the water–oil interface when the viscosity of silicone oil is 0.096 Pa·s. (**a**) Polypropylene particles; (**b**) Al_2_O_3_ particles.

When the viscosity of silicone oil is 0.096 Pa·s, the model calculation results are shown in Figure 8. The movement patterns of the two particles are consistent with the results when the viscosity of silicone oil is 0.048 Pa·s. The difference is that the polypropylene particles and Al_2_O_3_ particles of 2 mm diameter change from the pass type to the remain type; they cannot pass the water–oil interface to enter the oil layer. The final dimensionless displacement of polypropylene particles of 2 mm diameter is about 1.4R, while the final dimensionless displacement of Al_2_O_3_ particles of 2 mm diameter is only about 0.6R, which decreases significantly. At the same time, the time required for the two particles to pass the water–oil interface also slightly increases. The above difference may be attributed to the increase in viscosity of silicone oil, the value of cosθ_PWO_ decreases, and the overall wettability of the three phase decreases. As a result, the motion of particles at the water–oil interface is more resistant, and it is more difficult to pass the water–oil interface into the oil layer. According to the slope of the dimensionless displacement curve, it can be seen that as the viscosity increases, the slope decreases, that is, the velocity of particle movement decreases.

Comparing the dimensionless displacement curves of the two particles under different viscosity conditions, it can be concluded that: (1) the diameter of the particle has a significant effect on the time required for the particle to pass the water–oil interface, but the viscosity has a small effect; (2) the viscosity of silicone oil has a significant influence on the critical size of particles that can pass the water–oil interface; (3) the increase in viscosity will lead to the decrease of the overall wettability between particles, water, and oil and the decrease of the final position (dimensionless displacement) of particles remaining at the water–oil interface; and (4) the increase in viscosity will lead to the decrease in particle floating velocity.

### 3.3. Results of the Water Model

#### 3.3.1. Analysis of the Particle Floating Process

**Figure 9:**
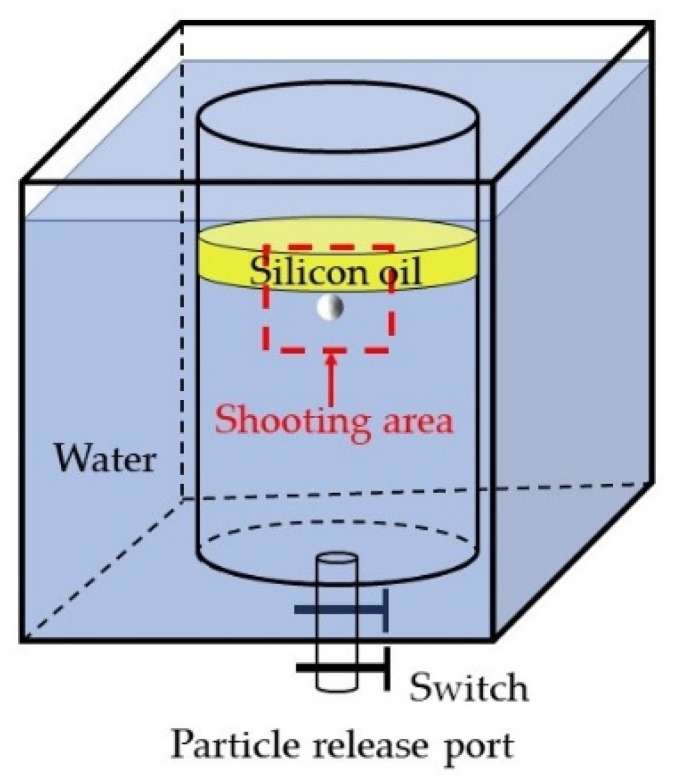
Schematic diagram of the photographing area.

The particles were statically released from the particle release port at the bottom of the container, and the overall height of the water surface was 50 cm. When particles floated up and approached the water–oil interface, a high-speed camera was used to record the motion process of particles under the same field of view. The shooting area was the red dotted box as shown in Figure 9, and the shooting time was 10 s.

**Figure 10:**
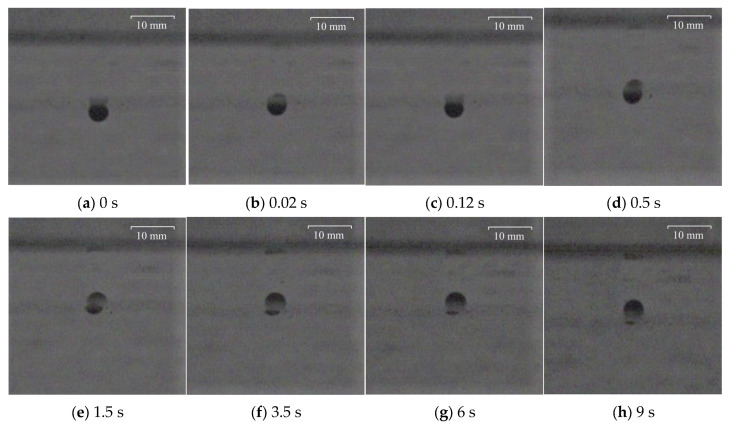
Motion process of polypropylene particle with a diameter of 3.98 mm at the water–oil interface with a silicone oil viscosity of 0.048 Pa·s: (**a**) 0 s, (**b**) 0.02 s, (**c**) 0.12 s, (**d**) 0.5 s, (**e**) 1.5 s, (**f**) 3.5 s, (**g**) 6 s, (**h**) 9 s.

**Figure 11:**
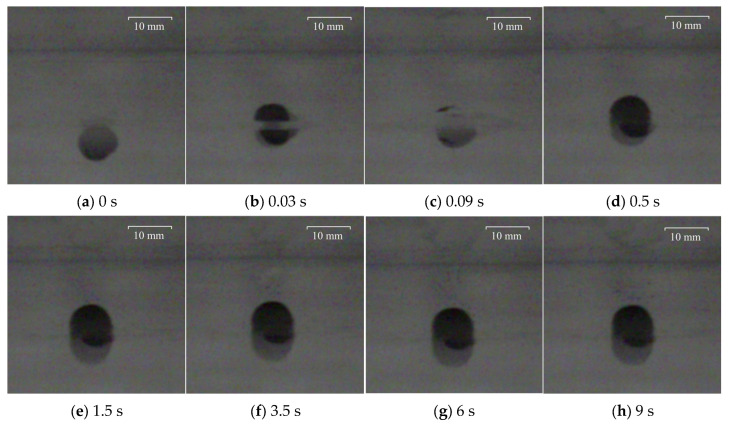
Motion process of Al_2_O_3_ particle with diameter of 4.26 mm at the water–oil interface with a silicone oil viscosity of 0.048 Pa·s: (**a**) 0 s, (**b**) 0.03 s, (**c**) 0.09 s, (**d**) 0.5 s, (**e**) 1.5 s, (**f**) 3.5 s, (**g**) 6 s, (**h**) 9 s.

**Figure 12:**
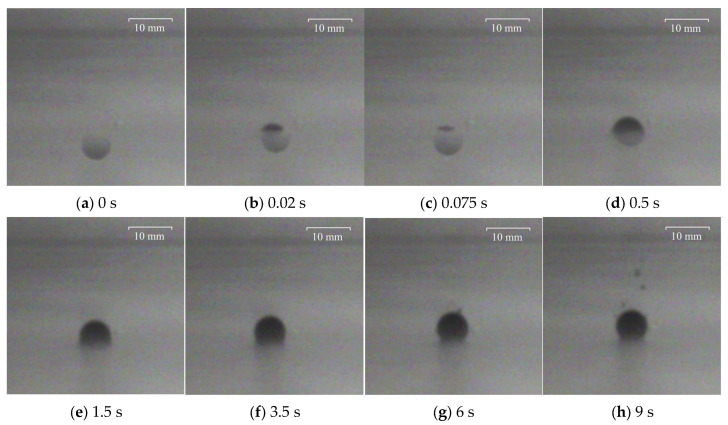
Motion process of polypropylene particle with diameter of 3.98 mm at the water–oil interface with a silicone oil viscosity of 0.096 Pa·s: (**a**) 0 s, (**b**) 0.02 s, (**c**) 0.075 s, (**d**) 0.5 s, (**e**) 1.5 s, (**f**) 3.5 s, (**g**) 6 s, (**h**) 9 s.

**Figure 13:**
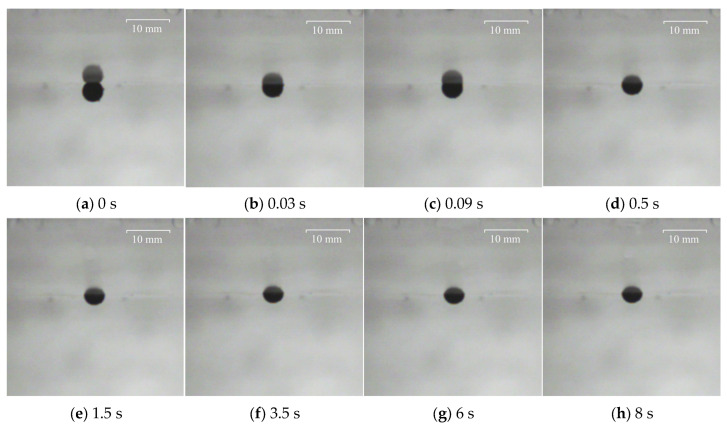
Motion process of Al_2_O_3_ particle with diameter of 4.26 mm at the water–oil interface with a silicone oil viscosity of 0.096 Pa·s: (**a**) 0 s, (**b**) 0.03 s, (**c**) 0.09 s, (**d**) 0.5 s, (**e**) 1.5 s, (**f**) 3.5 s, (**g**) 6 s, (**h**) 8 s.

To facilitate observation, the larger polypropylene particle with a diameter of 3.98 mm and the Al_2_O_3_ particle with a diameter of 4.26 mm were captured and recorded at the particular time at the water–oil interface with different viscosities of silicone oil. The results are shown in Figure 10, Figure 11, Figure 12 and Figure 13, where 0 s indicates the moment that particles float up and contact the water–oil interface.

As can be seen from Figure 10, after the polypropylene particle with a diameter of 3.98 mm contacts the water–oil interface, it reaches the highest position for the first time after 0.02 s. Then, there is a rebound phenomenon. After 0.1 s, it reaches the lowest point of the rebound and begins to slowly float, reaching the final highest position of the floating at about 3.5 s. It can be seen from Figure 11 that the time for Al_2_O_3_ particles with a diameter of 4.26 mm to reach the highest point of first floating is 0.03 s, which is slightly longer than the floating time of polypropylene particles. However, the rebound time of Al_2_O_3_ particles is only 0.06 s, which is slightly shorter than that of polypropylene particles. Then, the Al_2_O_3_ particles slowly floated up again, reaching the highest floating position at about 1.5 s.

To explore whether the viscosity of silicone oil had an effect on the motion of particles at the water–oil interface, the movement process of the above two particles at the water–oil interface with a silicone oil viscosity of 0.096 Pa·s was also studied. The experimental results show that the increase in silicone oil viscosity does not affect the overall motion of the two particles at the water–oil interface. However, the time for the polypropylene particle to rebound to the lowest position changes to 0.04 s, and the time of floating to the final highest position extends to about 6 s. The time of Al_2_O_3_ particle rebound to the lowest position changes to 0.05 s, and the time of floating to the final highest position extends to about 3.5 s.

When the viscosity of silicone oil is 0.048 Pa·s and 0.096 Pa·s, there are differences in the movement process of particles after moving to the water–oil interface. When the viscosity of the silicone oil is 0.096 Pa·s, the time for the particles to rebound to the lowest point is significantly reduced, and the time for the particles to rise again to the highest point is significantly longer. This shows that the increase in silicone oil viscosity increases the movement resistance of particles at the water–oil interface, thereby prolonging the time for particles to float to the highest position.

#### 3.3.2. Analysis of Experimental Results of Dimensionless Displacement of Particles

The high-speed video target tracking and measuring software VL 3.0.1398 was used to capture and analyze the captured video. The coordinates of particles in each frame of the video can be obtained by establishing the coordinate system, and the actual motion displacement of particles can be obtained by converting the pixel calibration size. To simplify the analysis, the dimensionless displacement (the ratio of the actual displacement of the particle to the diameter of the particle) of the particle was used to represent the position of the particle during its motion. The water level in this section of this part was 50 cm, and the experimental results are as follows.

**Figure 14:**
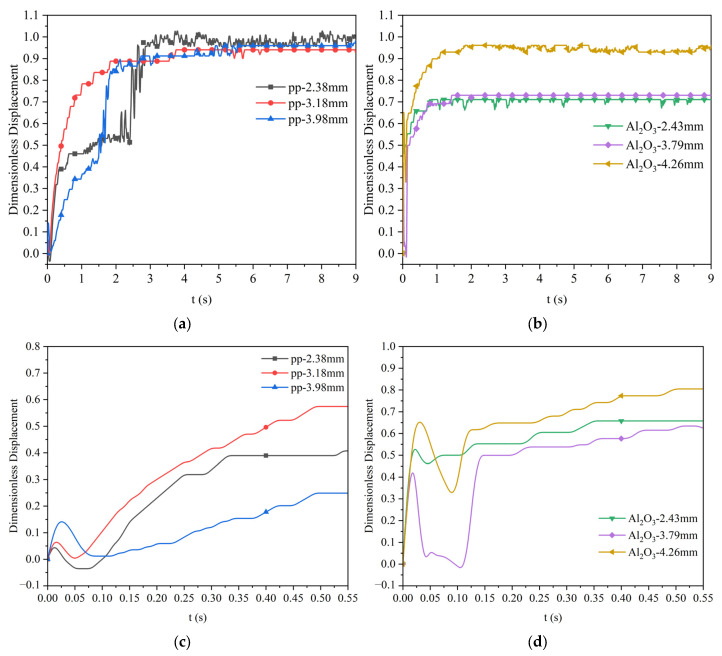
Dimensionless displacement curve of polypropylene and Al_2_O_3_ particles at the water–oil interface with a silicone oil viscosity of 0.048 Pa·s: (**a**) polypropylene particles; (**b**) Al_2_O_3_ particles; (**c**) enlarged diagram of polypropylene particles; (**d**) enlarged diagram of Al_2_O_3_ particles.

When the viscosity of the silicone oil is 0.048 Pa·s, the dimensionless displacement of polypropylene particles and Al_2_O_3_ particles of different particle sizes moving at the water–oil interface as a function of time is as shown in Figure 14. The figure shows the movement displacement of three polypropylene particles with different particle sizes and three Al_2_O_3_ particles with different particle sizes after reaching the water–oil interface (Z = 0). From the figure, it is more intuitive to observe the motion process of particles floating up, then rebounding, and finally floating up slowly until they reach the final highest position after reaching the water–oil interface. The two particles cannot completely pass the water–oil interface into the oil layer; the final dimensionless displacement of polypropylene particles with three different sizes is about 0.95, while the final dimensionless displacement of Al_2_O_3_ particles with a larger size of 4.26 mm can reach about 0.9. The final dimensionless displacement of Al_2_O_3_ particles with a smaller size of 2.43 mm and 3.79 mm is only about 0.7. Figure 14c,d show the enlarged version of the dimensionless displacement of the two particles just after contacting the interface (before 0.55 s). It can be seen from the figure that after the two particles reach the interface, and they first float up and then rebound at 0.02–0.03 s, finally slowly floating up to the oil layer.

**Figure 15:**
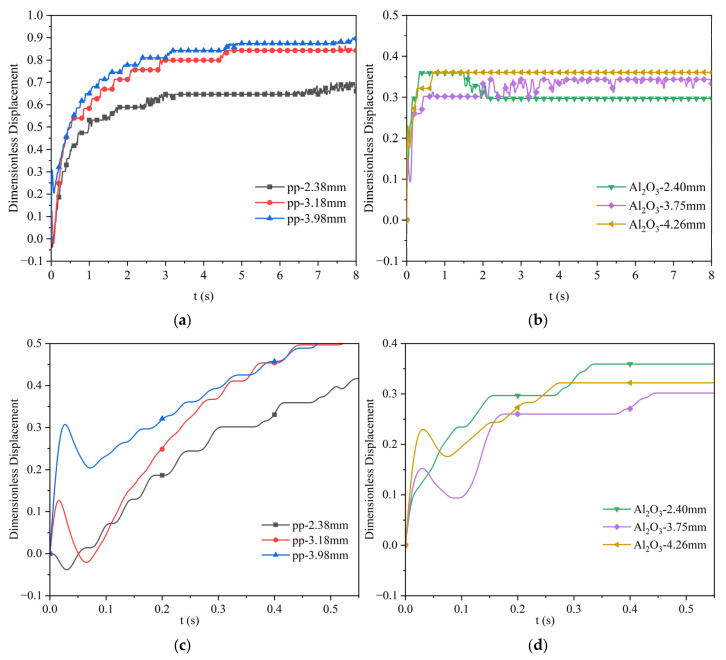
Dimensionless displacement curve of polypropylene and Al_2_O_3_ particles at the water–oil interface with a silicone oil viscosity of 0.096 Pa·s: (**a**) polypropylene particles; (**b**) Al_2_O_3_ particles; (**c**) enlarged diagram of polypropylene particles; (**d**) enlarged diagram of Al_2_O_3_ particles.

When the viscosity of the silicone oil is 0.096 Pa·s, the dimensionless displacement of polypropylene particles and Al_2_O_3_ particles of different particle sizes moving at the water–oil interface as a function of time is as shown in Figure 15. This figure shows that the final dimensionless displacement of polypropylene particles of 2.38 mm significantly decreases to about 0.6. In comparison, the values of polypropylene particles of 3.18 mm and 3.98 mm slightly decrease to about 0.8. The final dimensionless displacement of the three different sizes of Al_2_O_3_ particles significantly decreases, and the value is about 0.35. Figure 15c,d are enlarged versions of the dimensionless displacement diagrams of the two particles when they first come into contact with the interface (before 0.55 s). Similar to Figure 14c,d, after the two particles reach the interface, they first float up, then rebound, and finally slowly float up to the oil layer. The difference when comparing the oil with a lower viscosity case is that the rebound at the initial stage cannot be observed for small particles.

From the above analysis, the final dimensionless displacement of polypropylene particles is generally higher than that of Al_2_O_3_ particles. This is due to the better wettability of polypropylene particles.

**Figure 16:**
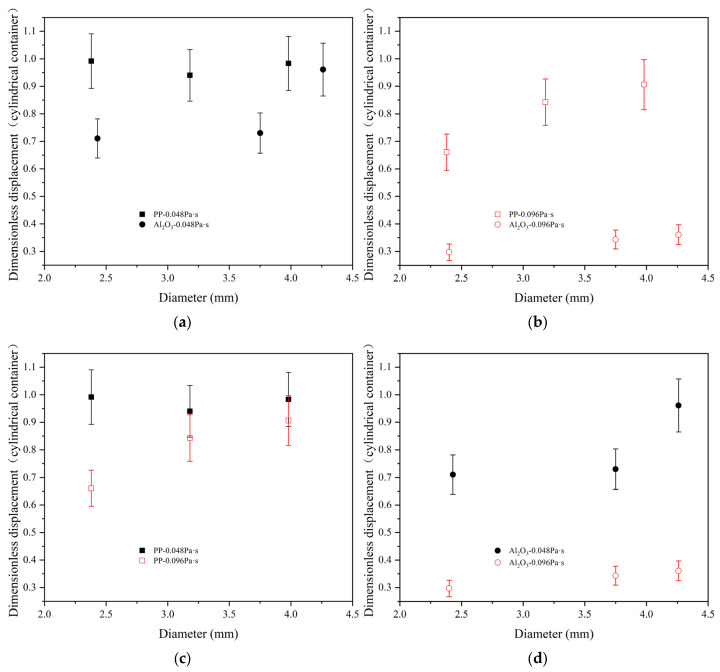
Dimensionless displacement comparison of particles in a cylindrical container. (**a**) Comparison when the viscosity of silicone oil is 0.048 Pa·s; (**b**) comparison when the viscosity of silicone oil is 0.096 Pa·s; (**c**) comparison of different viscosity of oil for polypropylene particles; (**d**) comparison of different viscosity of oil for Al_2_O_3_ particles.

It can be seen from Figure 16 that the dimensionless displacement of polypropylene particles and Al_2_O_3_ particles increases with the increase in particle diameter. According to Figure 16a,b, when the silicone oil viscosity is 0.048 Pa·s, the dimensionless displacement of polypropylene particles is about 1.0, and the dimensionless displacement of polypropylene particles does not change significantly with diameter. The dimensionless displacement of Al_2_O_3_ particles is 0.7 to 1.0. When the silicone oil viscosity is 0.096 Pa·s, the dimensionless displacement of polypropylene particles is about 0.65 to 0.9, and the dimensionless displacement of Al_2_O_3_ particles is between 0.3 and 0.4. As the silicone oil viscosity increases from 0.048 Pa·s to 0.096 Pa·s, the dimensionless displacement of both polypropylene particles and Al_2_O_3_ particles decreases. According to Figure 16c, when the silicone oil viscosity is 0.048 Pa·s, the dimensionless displacement of polypropylene particles is 0.95 to 1.0. When the silicone oil viscosity is 0.096 Pa·s, the dimensionless displacement of polypropylene particles is 0.65 to 0.9. After the silicone oil viscosity increases, the dimensionless displacement of polypropylene particles slightly decreases. According to Figure 16d, when the silicone oil viscosity is 0.048 Pa·s, the dimensionless displacement of Al_2_O_3_ particles is 0.70 to 0.95. When the silicone oil viscosity is 0.096 Pa·s, the dimensionless displacement of Al_2_O_3_ particles is 0.3 to 0.35. After the silicone oil viscosity increases, the dimensionless displacement of Al_2_O_3_ particles significantly decreases. For the Al_2_O_3_ particle with a diameter of 4.26 mm, the dimensionless displacement of Al_2_O_3_ particles in the oil layer decreases by about 0.6 when the silicone oil viscosity increases from 0.048 Pa·s to 0.096 Pa·s. For the polypropylene particles with good wettability, the increase in silicone oil viscosity has a non-significant effect on the dimensionless displacement. For the polypropylene particle with a diameter of 3.98 mm, the dimensionless displacement of polypropylene particles in the oil layer decreases by about 0.05 when the silicone oil viscosity increases from 0.048 Pa·s to 0.096 Pa·s. The viscosity has a negligible effect on the dimensionless displacement of larger polypropylene particles. For Al_2_O_3_ particles with poor wettability, the dimensionless displacement of the particles significantly decreases when the viscosity of silicone oil increases. However, when considering the two parameters of particle wettability and silicone oil viscosity, the effect of wettability on dimensionless displacement is greater than that of silicone oil viscosity.

#### 3.3.3. Analysis of Velocity Variation during Particle Motion

To further explore the movement mechanisms of particles during the process of floating, the velocity variation of particles as a function of time was analyzed. The particle’s velocity was obtained by differentiating the actual displacement curve of the particles. In the velocity curve, 0.22 s corresponds to the time of the particle’s contact with the water–oil interface, namely the t = 0 s in the dimensionless displacement curve. This time shift treatment is designed to show the velocity before attachment of the interface.

**Figure 17:**
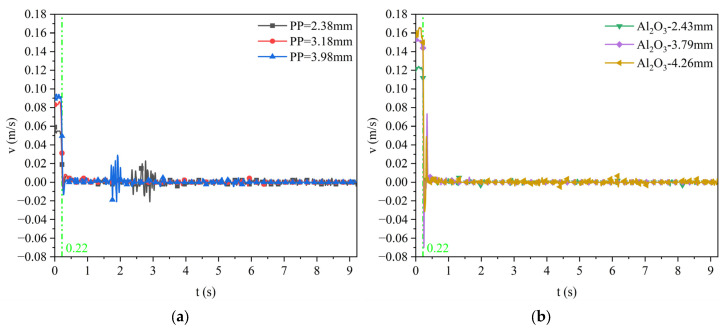

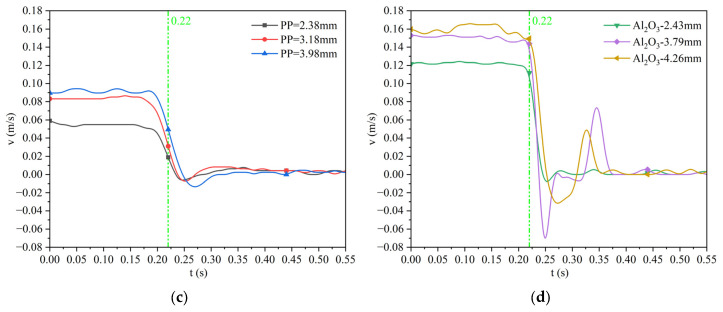
The particles floating velocity curve when the viscosity of silicone oil is 0.048 Pa·s: (**a**) polypropylene particles; (**b**) Al_2_O_3_ particles; (**c**) enlarged diagram of polypropylene particles; (**d**) enlarged diagram of Al_2_O_3_ particles.

For the silicone oil viscosity of the 0.048 Pa·s case, Figure 17 shows the velocity variation curve of polypropylene particles and Al_2_O_3_ particles with different sizes when they approach the water–oil interface until they finally remain at the water–oil interface. Among them, Figure 17c,d are the velocity change diagrams of the first 0.5 s. It can be seen from the figure that the terminal (maximum) velocity of polypropylene particles with a diameter of 2.38 mm, 3.18 mm, and 3.98 mm is about 0.058 m/s, 0.082 m/s, and 0.093 m/s, respectively. The terminal velocity of Al_2_O_3_ particles with a diameter of 2.43 mm, 3.79 mm, and 4.26 mm is about 0.122 m/s, 0.152 m/s, and 0.163 m/s, respectively. It indicates that the larger the diameter of the same kind of particle, the larger the terminal velocity; the particle has reached the maximum velocity before contacting with the water–oil interface and then decelerates close to the water–oil interface. The density of Al_2_O_3_ particles is smaller than that of polypropylene particles, but the terminal velocity of Al_2_O_3_ particles is larger.

**Figure 18:**
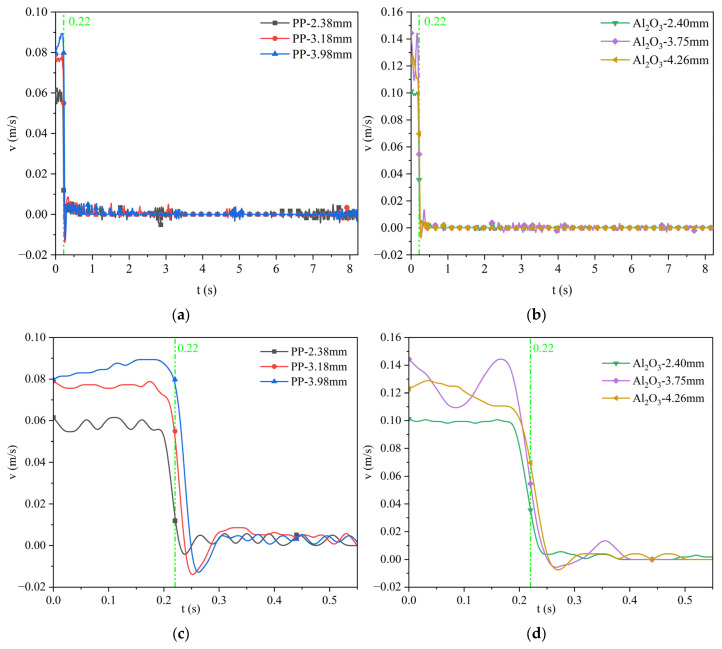
The particle-floating velocity curve when the viscosity of silicone oil is 0.096 Pa·s: (**a**) polypropylene particles; (**b**) Al_2_O_3_ particles; (**c**) enlarged diagram of polypropylene particles; (**d**) enlarged diagram of Al_2_O_3_ particles.

For the silicone oil viscosity of the 0.096 Pa·s case, Figure 18 shows the velocity variation curve of polypropylene particles and Al_2_O_3_ particles with different sizes when they approach the water–oil interface until they finally remain at the water–oil interface. Among them, Figure 18c,d are the velocity change diagrams of the first 0.5 s. It can be seen from the figure that the terminal velocity of polypropylene particles with a diameter of 2.38 mm, 3.18 mm, and 3.98 mm is about 0.055 m/s, 0.076 m/s, and 0.089 m/s, respectively. The terminal velocity of Al_2_O_3_ particles with a diameter of 2.40 mm, 3.75 mm, and 4.26 mm is about 0.102 m/s, 0.128 m/s, and 0.145 m/s, respectively. Compared with Figure 17, as the viscosity of silicone oil increases, the terminal velocity of all particles decreases. In other words, as the viscosity of silicone oil increases, the resistance for the motion of particles when approaching the water–oil interface increases.

**Figure 19:**
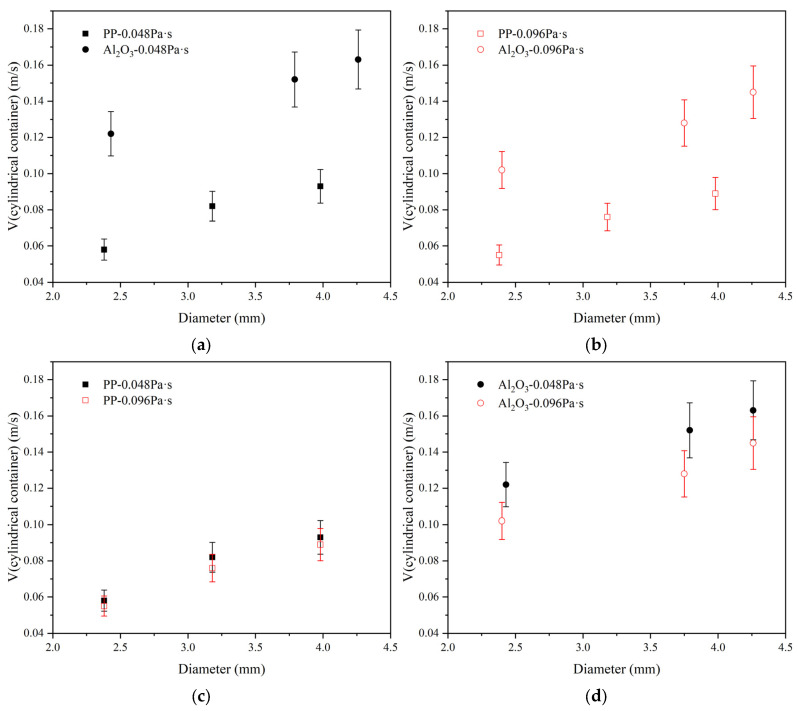
Terminal velocity comparison of particles in a cylindrical container. (**a**) Comparison when the viscosity of silicone oil is 0.048 Pa·s; (**b**) comparison when the viscosity of silicone oil is 0.096 Pa·s; (**c**) comparison of different viscosities of oil for polypropylene particle; (**d**) comparison of different viscosities of oil for Al_2_O_3_ particles.

The comparison of terminal velocities of the two particles is shown in Figure 19. According to Figure 19a,b, it can be seen that the terminal velocity of Al_2_O_3_ particles is higher than that of polypropylene particles. The difference of terminal velocity between two particles is decreased for the large viscosity of silicone oil case. As can be seen from Figure 19c,d, as the viscosity of silicone oil increases, the terminal velocity of both particles decreases, and the decreased amplitude of Al_2_O_3_ particles is greater than that of polypropylene particles. Particle diameter influences the terminal velocity. The larger the particle diameter, the larger the terminal velocity.

#### 3.3.4. Effect of Floating Distance of Particles on the Motion at the Water–Oil Interface

To explore whether the floating distance of particles had an effect on the motion process at the water–oil interface, the water level was reduced to 30 cm. The studied case was polypropylene particles with different sizes, and the viscosity of silicone oil was 0.048 Pa·s.

**Figure 20:**
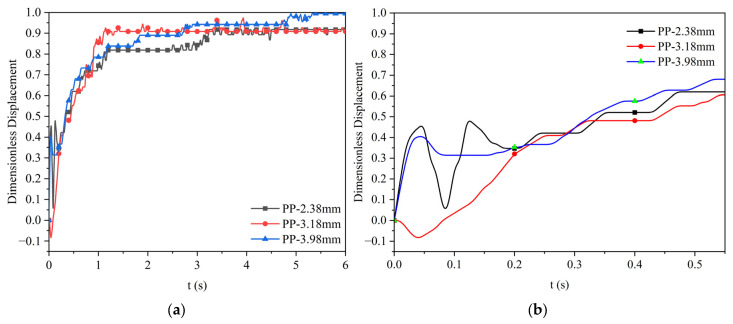
The dimensionless displacement curve of polypropylene particles at the water–oil interface when the viscosity of silicone oil is 0.048 Pa·s (water level is 30 cm). (**a**) Dimensionless displacement curve of polypropylene particles; (**b**) enlarged diagram of polypropylene particles.

**Figure 21:**
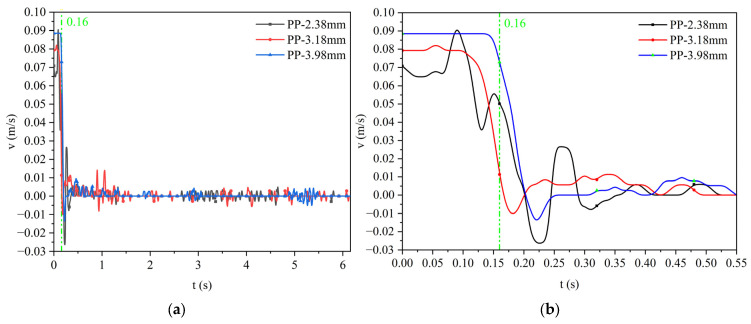
The polypropylene particles floating velocity curve when the viscosity of silicone oil is 0.048 Pa·s (water level is 30 cm). (**a**) Polypropylene particles floating velocity curve; (**b**) enlarged diagram of polypropylene particles.

**Figure 22:**
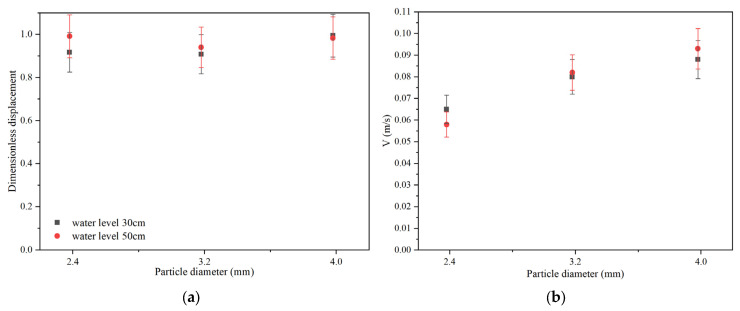
Comparison of experimental results of polypropylene particles at different water levels. (**a**) Dimensionless displacement; (**b**) terminal velocity.

The dimensionless displacement and velocity change curves of polypropylene particles are shown in Figure 20 and Figure 21, respectively. The comparison of experimental results between the water level of 30 cm and 50 cm is shown in Figure 22. It can be seen from Figure 22 that the final dimensionless displacements of three different diameters of polypropylene particles are around 0.917, 0.908, and 0.995, respectively. Compared with the case where the liquid level is 50 cm, the dimensionless displacement of particle with a diameter of 2.38 mm and 3.18 mm is less than the corresponding results, while the particle with a diameter of 3.98 mm is slightly higher than the corresponding result. The particle dimensionless displacement deviation proportions for the particles with diameters of 2.38 mm, 3.18 mm, and 3.98 mm are 7.5%, 3.3%, and 1.1%, respectively. For the liquid level in the 30 cm case, the terminal velocities of the three sizes are around 0.065 m/s, 0.080 m/s, and 0.088 m/s, respectively. Compared with the case where the liquid level is 50 cm, the particle terminal velocity of particle with a diameter of 2.38 mm is larger than the corresponding result, while the velocities of particles with diameters of 3.18 mm and 3.98 mm are slightly smaller than the corresponding results. The deviation proportions of the three sizes are 9.8%, 2.4%, and 5.3%, respectively. According to the deviation ratio analysis of the experimental results, in this experiment, the height of the water level did not significantly affect the movement of the particles. At the same time, it can be concluded from Section 3.3.3 that the particles have reached the maximum velocity when they reach the water–oil interface.

### 3.4. Discussions

In this study, silicone oils of 0.048 Pa·s and 0.096 Pa·s were used in a static cylindrical container to simulate the slag layer in the metallurgical container, and polypropylene particles and Al_2_O_3_ particles of different sizes were released from the bottom of the container. This study simulates the movement behavior of inclusions at the steel–slag interface. Based on Newton’s second law, a particle floating motion model is constructed to verify the water model experiment.

(1) The viscosity of silicone oil affects the dimensionless displacement and terminal velocity of particles. When the viscosity of the silicone oil is 0.048 Pa·s, the final dimensionless displacement of the polypropylene particles is about 0.95. When the viscosity of the silicone oil is 0.096 Pa·s, the dimensionless displacement of the same polypropylene particles is about 0.6 to 0.8. It can be found that the displacement of particles significantly decreases as the viscosity of silicone oil increases. When the viscosity of silicone oil is 0.048 Pa·s, the terminal velocities of polypropylene particles with diameters of 2.38 mm, 3.18 mm, and 3.98 mm are 0.058 m/s, 0.082 m/s, and 0.093 m/s, respectively. When the viscosity of the silicone oil increases to 0.096 Pa·s, the terminal velocities of the same sized particles are 0.055 m/s, 0.076 m/s, and 0.089 m/s, respectively. It can be clearly seen that the terminal velocity of the particles slightly decreases as the viscosity of silicone oil increases. Mathematical models have shown the same results. When the viscosity of silicone oil is 0.048 Pa·s, the dimensionless displacement of all particles is greater than two, and the particles can pass through the water–oil interface and enter the oil layer. When the viscosity of the silicone oil increases to 0.096 Pa·s, the smaller particles cannot be removed from the oil layer. The result is due to the damping effect of the viscous oil layer on the movement of particles.
(2) The particle diameter also affects the dimensionless displacement and terminal velocity of the particle. When the viscosity of silicone oil is 0.048 Pa·s, the final dimensionless displacement of Al_2_O_3_ particles with a diameter of 4.26 mm is around 0.95. The dimensionless displacement of Al_2_O_3_ particles with diameters of 2.43 mm and 3.79 mm is only about 0.7. The larger the particle diameter, the larger the dimensionless displacement. For the terminal velocity, under the same viscosity conditions, the terminal velocities of Al_2_O_3_ particles with diameters of 2.40 mm, 3.79 mm, and 4.26 mm are 0.122 m/s, 0.152 m/s, and 0.163 m/s, respectively. The larger the particle diameter, the greater the terminal velocity. The polypropylene particles also showed similar tendency. Mathematical model results also show that the larger the particle diameter, the greater the velocity. However, Zhou [71] found that, in water model experiments, the particle diameter has little effect on dimensionless displacement, which is different from the result of this article.
(3) For different types of particles, there are certain differences in the dimensionless displacement and terminal velocity of the two particles. Water model experiments show that when the viscosity of silicone oil is 0.096 Pa·s, the dimensionless displacements of polypropylene particles with a diameter of 2.38 mm and Al_2_O_3_ particles with a diameter of 2.43 mm are 0.6 and 0.35, respectively. The dimensionless displacement of polypropylene particles is larger than that of Al_2_O_3_ particles of the same diameter. This is because the cosθ_PWO_ of polypropylene particles is smaller than that of Al_2_O_3_ particles, resulting in the overall three-phase wettability of polypropylene particles being better than that of Al_2_O_3_ particles. The terminal velocities of 2.38 mm and 3.98 mm polypropylene particles are 0.055 m/s and 0.089 m/s, respectively, and the terminal velocities of Al_2_O_3_ particles with a diameter of 2.40 mm and 4.26 mm are 0.102 m/s and 0.145 m/s, respectively. The terminal velocity of polypropylene particles is smaller than that of Al_2_O_3_ particles of the same diameter, which may be due to the fact that the density of Al_2_O_3_ particles is smaller. The mathematical model results show that the dimensionless displacement curve of Al_2_O_3_ particles has a larger slope and rises faster, which is consistent with the experimental results of the water model.
(4) To explore whether the liquid level height affects the particle behavior, this article conducted experiments at two different liquid level heights of 30 cm and 50 cm. For particles with three different diameters at two different liquid level heights, the overall deviation of dimensionless displacement is less than 7.5% and this deviation is decreased when the diameter of the particle is increased (3.3% and 1.1% for particle diameters of 3.18 mm and 3.98 mm). The overall deviation of terminal velocities is less than 9.8%. The particle with a diameter of 3.18 mm showed a deviation of 2.4%. It can be considered that when the liquid level is 30 cm or 50 cm, the particles have reached the maximum velocity during the floating process. Meanwhile the liquid level height was 25 cm in the research of Ferchichi and Duval [56], and their study fluid was glycerol and silicone oil. The liquid level height in water–oil systems were 32 cm in the study by Zhou et al. [59], 40 cm in the study by Zhu et al. [60], and 20 cm in study by Nishibata et al. [61], respectively. The even lower height is not considered in this study and could be studied in a future work.

Recently, Saito [72] organized a research group in Japan and focused on the visualization and sensing of slag for a better understanding of multi-phase metal flow. A number of research studies on oil–particle systems have been carried out. Similar work on the settling of particles in a suspension oil system were studied by Shimasaki et al. [73,74]. It must be mentioned that this study was conducted under static conditions, which is different from the floating movement of inclusions in actual metallurgical reactors. Nonetheless, this study provides a fundamental basis for the inclusion removal process. Future research should consider simulating a more realistic flow field in reactors to further explore the inclusion removal process in metallurgical reactors.

## 4. Conclusions

In this paper, the motion behavior at the water–oil interface of polypropylene and Al_2_O_3_ particles with different sizes at different viscosity of silicone oil was systematically analyzed, and the following conclusions can be drawn:

(1) When the viscosity of silicone oil increases from 0.048 Pa·s to 0.096 Pa·s, both the dimensionless displacement and terminal velocity of the particles decreases. When the viscosity of the silicone oil is 0.048 Pa·s, the final dimensionless displacements of polypropylene particles of three different particle sizes are all about 0.95, and their terminal velocities range from 0.058 m/s to 0.093 m/s. The final dimensionless displacement of Al_2_O_3_ particles of three different particle sizes is around 0.7 to 0.9, and their terminal velocity is between 0.122 m/s and 0.163 m/s. When the viscosity of the silicone oil increases to 0.096 Pa·s, the final dimensionless displacement of the polypropylene particles of three different particle sizes is about 0.6 to 0.8, and the terminal velocity decreases slightly to the range of 0.055 m/s to 0.089 m/s. The final dimensionless displacement of Al_2_O_3_ particles of three different particle sizes is only about 0.35, and their terminal velocities range from 0.102 m/s to 0.145 m/s. The mathematical model results also show that as the viscosity of silicone oil increases, the dimensionless displacement and terminal velocity of the particles decrease.
(2) For the same type of particle, the dimensionless displacement and terminal velocity increases as the diameter increases. When the viscosity of the silicone oil is 0.048 Pa·s, the final dimensionless displacement of the Al_2_O_3_ particles with diameter of 2.43 mm and 3.79 mm is about 0.7, and the final dimensionless displacement of the Al_2_O_3_ particle with a diameter of 4.26 mm is about 0.9. This tendency also works in the case with other viscosities. The mathematical model results also show that the larger the particle diameter, the greater the terminal velocity, which is consistent with the water model experimental results.
(3) When comparing two types of particles, the dimensionless displacement of polypropylene particles of the same diameter is larger than that of Al_2_O_3_ particles, and the terminal velocity is smaller than that of Al_2_O_3_ particles. When the viscosity of silicone oil is 0.096 Pa·s, the dimensionless displacements of polypropylene particles with a diameter of 2.38 mm and Al_2_O_3_ particles with a diameter of 2.43 mm are 0.6 and 0.35, respectively. The terminal velocities of polypropylene particles with diameters of 2.38 mm and 3.98 mm are 0.055 m/s and 0.089 m/s, respectively, and the terminal velocities of Al_2_O_3_ particles with diameters of 2.40 mm and 4.26 mm are 0.102 m/s and 0.145 m/s, respectively. The dimensionless displacements of polypropylene particles are larger than that of Al_2_O_3_ particles, while the terminal velocities of polypropylene particles are smaller than that of Al_2_O_3_ particles of the same diameter. The mathematical model results show that the dimensionless displacement curve of Al_2_O_3_ particles has a larger slope and faster rising tendency, which is consistent with the experimental results of the water model.
(4) Data from the water model show that when the liquid level changes from 30 cm to 50 cm, the dimensionless displacement and terminal velocity of the same particles in silicone oil show only slight differences. The dimensionless displacement and terminal velocity deviation ratios are both less than 10%.

## Figures and Tables

**Table 1:** Physical parameters of the materials used in the experiment.

Material	Diameter /mm	Density /kg·m^−3^	Surface Tension /N·m^−1^	Viscosity /Pa·s
Water	-	1000	0.0728	0.001
Silicone oil	-	960/963	0.021	0.048/0.096
Polypropylene	2.38/3.18/3.98	910	0.031	-
Hollow Al_2_O_3_	2–5	710	0.65	-

**Table 2:** Interfacial tension parameter of the water model.

Material	Viscosity/Pa·s	σ_PW_/N·m	σ_PO_/N·m	σ_WO_/N·m	cosθ_PWO_
Polypropylene	0.048	0.634	0.610	0.041	0.585
	0.096	0.634	0.615	0.055	0.345
Al_2_O_3_	0.048	0.614	0.630	0.041	−0.390
	0.096	0.614	0.635	0.055	−0.382

## Data Availability

Data are contained within the article.
